# Inaccurate Multilocus Sequence Typing of *Acinetobacter baumannii*

**DOI:** 10.3201/eid2501.180374

**Published:** 2019-01

**Authors:** Santiago Castillo-Ramírez, Lucía Graña-Miraglia

**Affiliations:** Programa de Genómica Evolutiva, Centro de Ciencias Génomicas, Universidad Nacional Autónoma de México, Cuernavaca, Mexico

**Keywords:** genotyping, multilocus sequence typing, MLST, *Acinetobacter baumannii*, genome sequencing, bacteria

## Abstract

Multilocus sequence typing has been useful for genotyping pathogens in surveillance and epidemiologic studies. However, it cannot reflect the true relationships of isolates for species with very dynamic genomes. Using a robust genome phylogeny, we demonstrated the limitations of this method for typing *Acinetobacter baumannii*.

An adequate genotyping system is of paramount importance for infectious disease epidemiology. Two decades ago, the multilocus sequence typing (MLST) scheme was proposed as a genotyping method (*1*), and today, because of its reproducibility and portability, MLST schemes are available for many human pathogens (*2*). MLST has been instrumental in increasing understanding of the epidemiology and population structure of many bacteria. *Acinetobacter baumannii*, a major source of nosocomial infections, is no exception, and 2 MLST schemes (Oxford and Pasteur) have been established for this species (*3*,*4*). Each scheme uses just 7 loci and, therefore, only samples a small fraction of the chromosome, which could be a serious issue for genotyping species with highly variable genomes. Some studies have shown that *A. baumannii* has both high gene content variation (*5*) and substantial levels of recombination (*6*).

We revisited one of the most comprehensive genome datasets of *A. baumannii* (*5*) to construct a robust phylogeny to show that sequence type (ST) assignation in both MLST schemes does not reflect true relationships among isolates of this species. This dataset of >80 genomes covers 36 different STs according to the Oxford scheme (STox) and 19 different STs according to the Pasteur scheme (STp) (Appendix Table 1). We constructed a concatenated alignment of 574 orthologous genes and conducted statistical model selection as in a previous study (*5*) and, on that alignment, constructed a maximum-likelihood phylogeny by PhyML (*7*).

The 2 schemes showed different levels of resolution. Although in many instances a single STp had just 1 equivalent STox, 2 STs exist in the Pasteur scheme that encompass many Oxford STs (Appendix Figure, red branches). For instance, under the Pasteur scheme, STp2 represents >15 STs in the Oxford scheme and STp1 encompasses 5 STox. Thus, the Pasteur scheme seems to have considerably less resolution than the Oxford scheme to distinguish isolates. The Pasteur scheme’s lack of resolution was not insignificant, however. STp2 comprises 43 isolates (approximately half of our dataset) showing considerable levels of genetic variation according to our phylogeny, but according to this MLST scheme, they constitute just 1 genotype.

Many of the STs in either scheme formed coherent (monophyletic) groups in our phylogeny. However, we recorded some clear exceptions in which isolates from some STs did not form monophyletic groups, that is, isolates with the same ST did not cluster. The most striking case is STox208 (orange tips in the phylogeny), where there are 2 well-defined groups with several isolates each and an extra isolate not close to either of those well-defined groups. We also noted that the 2 STox455 isolates did not cluster and are located far apart on the tree (green tips). Additionally, 1 of the STox369 isolates did not fall within the ST369 group (blue tips). These 3 examples show that the Oxford MLST does not accurately reflect the relationships among the isolates. Also notable is that, although for the Oxford scheme 36 STs are represented in this dataset, only 16 of them have >2 isolates and therefore only in these STs could we detect problems with the clustering within any given ST. Thus, 3 of these 16 STs did not cluster the isolates properly inasmuch as these STs were polyphyletic. In summary, for the Oxford scheme we demonstrated that some STs form polyphyletic groups because 4 of the 7 loci have signals of recombination (Appendix Table 2), whereas for the Pasteur scheme, we noted a serious lack of resolution for some STs because the loci used only by this scheme have the lowest levels of genetic diversity (Appendix Table 2). Two previous studies noted problems with the MLST schemes for this species (*8*,*9*); nonetheless, neither was as extensive as our study, nor did they benchmark both schemes against a genome-based phylogeny.

In conclusion, we showed that the correct relationships among isolates cannot be recovered using either of the MLST schemes for *A. baumannii*. In addition, we highlighted the importance of using more powerful genotyping strategies when analyzing bacteria with highly dynamic genomes; in this regard, the ever-decreasing cost of genome sequencing will make this technology the perfect tool for genotyping bacterial species.

Appendix *Acinetobacter baumannii* genome dataset, measures of genetic diversity and recombination for both MLST schemes, and maximum-likelihood phylogeny depicting the relationships among isolates in study of multilocus sequence typing of *Acinetobacter baumannii*.
