# Supplementary material for: Inaccurate Multilocus Sequence Typing of Acinetobacter baumannii
Source: Emerg Infect Dis. 2019 Jan;25(1):186–7. doi: 10.3201/eid2501.180374 (PMC6302598; doi:10.3201/eid2501.180374)
Supplement: Appendix — Acinetobacter baumannii genome dataset, measures of genetic diversity and recombination for both MLST schemes, and maximum-likelihood phylogeny depicting the relationships among isolates in study of multilocus sequence typing of Acinetobacter baumannii. [file 18-0374-Techapp-s1.pdf]

Article DOI: https://doi.org/10.3201/eid2412.180374

Inaccurate Multilocus Sequence Typing of *Acinetobacter baumannii*

Appendix

Appendix Table 1. Genome dataset employed for this study*

Strain name	Assembly ID	Oxford scheme ST assignation	Pasteur scheme ST assignation
D1279779	GCA_000186665.4	942	267
D36	GCA_001399655.1	498	81
A85	GCA_002210065.1	781	1
ATCC-17978	GCA_000015425.1	112	437
AB031	GCA_000746605.1	1000	638
AB030	GCA_000746645.1	758	79
Ab04-mff	GCA_001077655.1	447	10
MDR-TJ	GCA_000187205.4	369	2
MDR-ZJ06	GCA_000226275.1	643	2
BJAB07104	GCA_000419385.1	368	2
BJAB0715	GCA_000419405.1	642	23
BJAB0868	GCA_000419425.1	218	2
ZW85–1	GCA_000505685.2	378	639
XH386	GCA_001026965.1	208	2
XH860	GCA_001573065.1	457	2
XH859	GCA_001573085.1	368	2
XH857	GCA_001573105.1	806	215
XH856	GCA_001573125.1	381	2
XH858	GCA_001578145.1	642	23
HRAB-85	GCA_001887305.1	208	2
XDR-BJ83	GCA_001902375.1	368	2
Ab6200	GCA_000814345.1	1161	464
AYE	GCA_000069245.1	231	1
R2090	GCA_001261895.2	942	267
CIP70–10	GCA_001457535.1	819	126
R2091	GCA_001517645.1	819	126
B8342	GCA_001077555.2	NA	NA
B8300	GCA_001077965.2	NA	NA
ACICU	GCA_000018445.1	437	2
NCGM-237	GCA_000828795.1	455	2
AB042	GCA_001941765.1	112	437
AC29	GCA_000695855.2	195	2
AC30	GCA_000307975.2	195	2
Ab3207	GCA_001636235.1	1321	422
AF-401	GCA_001896005.1	new	79
IOMTU-433	GCA_000828935.1	919	622
Ab1656–2	GCA_000188215.1	423	2
KBN10P02143	GCA_001514375.1	191	2
YU-R612	GCA_001543995.1	191	2
DU202	GCA_000498375.2	423	2
KAB01	GCA_001806345.1	451	2
KAB02	GCA_001806365.1	369	2
KAB03	GCA_001806385.1	451	2
KAB04	GCA_001806405.1	191	2
KAB05	GCA_001806425.1	369	2
KAB06	GCA_001806445.1	369	2
KAB07	GCA_001806465.1	191	2
KAB08	GCA_001806485.1	208	2
JBA13	GCA_002082625.1	191	2
CBA7	GCA_002082645.1	208	2
15A34	GCA_002082685.1	872	2
15A5	GCA_002082705.1	191	2
USA2	GCA_002082725.1	357	2
SSA6	GCA_002082745.1	357	2

Strain name	Assembly ID	Oxford scheme ST assignation	Pasteur scheme ST assignation
HWBA8	GCA_002082785.1	229	25
WKA02	GCA_002082805.1	447	10
USA15	GCA_002082825.1	491	1
SAA14	GCA_002082845.1	191	187
SSA12	GCA_002082865.1	191	2
SSMA17	GCA_002082885.1	191	2
ABH12O-A2	GCA_000761175.1	924	79
TYTH-1	GCA_000302575.1	455	2
TCDC-AB0715	GCA_000189735.2	218	2
A1	GCA_000830055.1	231	2
A1	GCA_000830055.1	231	2
AB307–0294	GCA_000021145.1	231	1
AB0057	GCA_000021245.1	207	1
LAC-4	GCA_000786735.1	447	10
ORAB01	GCA_000939415.2	208	2
AB5075-UW	GCA_000963815.1	945	1
AF-673	GCA_001895985.1	208	2
CMC-CR-MDR-Ab4	GCA_001922205.1	208	2
CMC-MDR-Ab59	GCA_001922225.1	208	2
CMC-CR-MDR-Ab66	GCA_001922245.1	208	2
ab736	GCA_002116925.1	931	52
BAL062	GCA_900088705.1	136	NA
AB34299	GCA_002009115.1	436	2
Ab11598	MSCZ00000000	758	156
Ab11536	MSCY00000000	758	156
Ab4113	MSDA00000000	758	156
Ab11502	MSCX00000000	758	156
Ab11551	MSDC00000000	758	156
Ab11547	MSDB00000000	758	156
Ab11606	MSDD00000000	758	156
Ab11510	CP018861/CP018862	758	156

*ID, identification; ST, sequence type.

Appendix Table 2. Measures of genetic diversity and recombination test for the loci of both multilocus sequence typing schemes for Acinetobacter baumannii

Gene	Scheme	Proportion of variable sites	Nucleotide diversity	PhiTest p value*
gdhB	Oxford	0.263	0.1060244	**2.83e-02**
gpi	Oxford	0.431	0.0798361	**0.00e+00**
gyrB	Oxford	0.047	0.0096987	**1.44e-09**
recA	Both	0.029	0.0044261	5.74e-02
cpn60	Both	0.024	0.0043936	**1.46e-02**
gltA	Both	0.022	0.0036340	2.15e-01
pyrG	Pasteur	0.025	0.0031657	**1.34e-03**
rpoD	Oxford	0.016	0.0031083	1.26e-01
rpoB	Pasteur	0.021	0.0029657	**3.50e-04**
rplB	Pasteur	0.010	0.0019185	1
fusA	Pasteur	0.010	0.0009114	1.89e-01

*Bold indicates statistical significance.

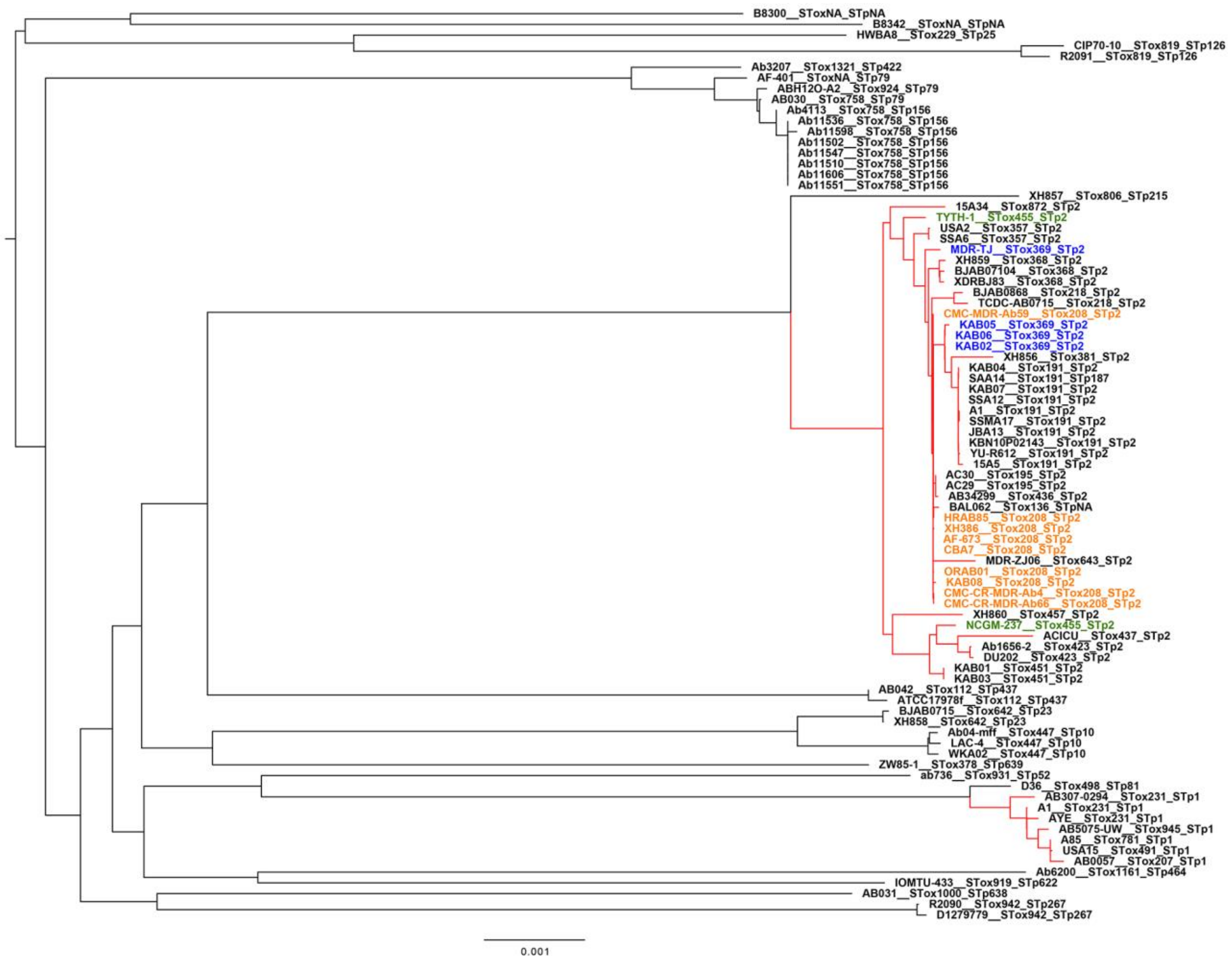

Appendix Figure. Maximum-likelihood phylogeny depicting the relationships among *Acinetobacter baumannii* isolates considered in this study. The phylogeny was constructed on the concatenated alignment of all the single gene families not showing recombination signals. Statistical model selection was conducted to determine the most adequate model, which was GTR+R+I. When possible, the sequence types in accordance with the Oxford scheme (STox) and the Pasteur scheme (STp) are shown. The red branches show cases of single STp that encompass many STox. Colored tips show polyphyletic groups under the Oxford scheme: blue, STox369; orange, STox208; green, STox455. Scale bar indicates nucleotide substitutions per site.
